# Genetic variation in insulin-like growth factor signaling genes and breast cancer risk among *BRCA1 *and *BRCA2 *carriers

**DOI:** 10.1186/bcr2414

**Published:** 2009-10-20

**Authors:** Susan L Neuhausen, Sean Brummel, Yuan Chun Ding, Christian F Singer, Georg Pfeiler, Henry T Lynch, Katherine L Nathanson, Timothy R Rebbeck, Judy E Garber, Fergus Couch, Jeffrey Weitzel, Steven A Narod, Patricia A Ganz, Mary B Daly, Andrew K Godwin, Claudine Isaacs, Olufunmilayo I Olopade, Gail Tomlinson, Wendy S Rubinstein, Nadine Tung, Joanne L Blum, Daniel L Gillen

**Affiliations:** 1 Department of Epidemiology, University of California Irvine, 224 Irvine Hall, Irvine, CA 92697, USA; 2 Department of Statistics, University of California Irvine, 2226 Bren Hall, Irvine, CA 92697 USA; 3 Division of Special Gynecology, Department of Obstetrics and Gynaecology, Medical University of Vienna, Waehringer Guertel 18-20, 1090 Vienna, Austria; 4 Department of Preventive Medicine and Public Health, Creighton University School of Medicine, 2500 California Plaza, Omaha, NE 68182, USA; 5 Department of Medicine, Medical Genetics, University of Pennsylvania School of Medicine, 351 BRB 2/3, 421 Curie Blvd, Philadelphia, PA 19104, USA; 6 Department of Biostatistics and Epidemiology Abramson Cancer Center University of Pennsylvania School of Medicine, 217 Blockley Hall, 423 Guardian Drive, Philadelphia, PA 19104, USA; 7 Department of Adult Oncology, Dana Farber Cancer Institute, 44 Binney Street, Boston, MA 02115, USA; 8 Mayo Department of Laboratory Medicine and Pathology, Mayo Clinic, 200 First Street SW, Rochester, MN 55905, USA; 9 Division of Clinical Cancer Genetics, Department of Population Sciences, City of Hope National Medical Center, 1500 East Duarte Road, Duarte, CA 91010, USA; 10 Women's College Research Institute, Women's College Hospital, 790 Bay Street, 7th Floor, Toronto, ON M5G 1N8, Canada; 11 UCLA Schools of Medicine and Public Health Division of Cancer Prevention & Control Research, Jonsson Comprehensive Cancer Center, 650 Charles Young Drive South, Room A2-125 CHS, Los Angeles, CA 90095-6900, USA; 12 Department of Clinical Genetics, Fox Chase Cancer Center, 333 Cottman Avenue, Philadelphia, PA 19111, USA; 13 Department of Medical Oncology, Fox Chase Cancer Center, 333 Cottman Avenue, Philadelphia, PA 19111, USA; 14 Department of Medicine and Oncology, Georgetown University, 3800 Reservoir Road, NW, Washington, DC 20007, USA; 15 Departments of Medicine and Human Genetics, University of Chicago, 5841 S. Maryland Avenue, MC2115, Chicago, IL 60637, USA; 16 Division of Pediatric Hematology Oncology, University of Texas Health Science Center at San Antonio, 7703 Floyd Curl Drive, San Antonio, TX 78229-3901, USA; 17 Department of Medicine, NorthShore University Health System, 1000 Central Street, Suite 620, Evanston, IL 60201, USA; 18 Department of Medical Oncology, Beth Israel Deaconess Medical Center, 330 Brookline Avenue, Boston, MA 02215, USA; 19 Department of Oncology, Baylor University Medical Center, 3535 Worth St. Suite 600, Dallas, TX 75246, USA

## Abstract

**Introduction:**

Women who carry mutations in *BRCA1 *and *BRCA2 *have a substantially increased risk of developing breast cancer as compared with the general population. However, risk estimates range from 20 to 80%, suggesting the presence of genetic and/or environmental risk modifiers. Based on extensive *in vivo *and *in vitro *studies, one important pathway for breast cancer pathogenesis may be the insulin-like growth factor (IGF) signaling pathway, which regulates both cellular proliferation and apoptosis. BRCA1 has been shown to directly interact with IGF signaling such that variants in this pathway may modify risk of cancer in women carrying BRCA mutations. In this study, we investigate the association of variants in genes involved in IGF signaling and risk of breast cancer in women who carry deleterious *BRCA1 *and *BRCA2 *mutations.

**Methods:**

A cohort of 1,665 adult, female mutation carriers, including 1,122 *BRCA1 *carriers (433 cases) and 543 *BRCA2 *carriers (238 cases) were genotyped for SNPs in *IGF1*, *IGF1 *receptor (*IGF1R*), IGF1 binding protein (*IGFBP1*, *IGFBP2, IGFBP5*), and IGF receptor substrate 1 (*IRS1*). Cox proportional hazards regression was used to model time from birth to diagnosis of breast cancer for *BRCA1 *and *BRCA2 *carriers separately. For linkage disequilibrium (LD) blocks with multiple SNPs, an additive genetic model was assumed; and for single SNP analyses, no additivity assumptions were made.

**Results:**

Among *BRCA1 *carriers, significant associations were found between risk of breast cancer and LD blocks in *IGF1R *(global *P *= 0.011 for LD block 2 and global *P *= 0.012 for LD block 11). Among *BRCA2 *carriers, an LD block in *IGFBP2 *(global *P *= 0.0145) was found to be associated with the time to breast cancer diagnosis. No significant LD block associations were found for the other investigated genes among *BRCA1 *and *BRCA2 *carriers.

**Conclusions:**

This is the first study to investigate the role of genetic variation in IGF signaling and breast cancer risk in women carrying deleterious mutations in *BRCA1 *and *BRCA2*. We identified significant associations in variants in *IGF1R *and *IRS1 *in *BRCA1 *carriers and in IGFBP2 in *BRCA2 *carriers. Although there is known to be interaction of *BRCA1 *and IGF signaling, further replication and identification of causal mechanisms are needed to better understand these associations.

## Introduction

Women who carry mutations in the *BRCA1 *and *BRCA2 *genes have a substantially increased risk of developing breast cancer and ovarian cancers as compared with the general population. Estimates of the age-specific risk attributable to mutations at these loci vary depending on the ascertainment scheme. The cumulative risks of breast cancer by age 70 were estimated to be 65% and 45% for *BRCA1 *and *BRCA2 *mutation carriers, respectively, in a meta-analysis of population-based studies [1] - as compared with 56 to 80% for *BRCA1 *and *BRCA2 *mutation carriers, respectively, in analyses based on families with multiple affected individuals [2-5].

Among *BRCA1 *and *BRCA2 *mutation carriers, there is considerable variability in both the age at diagnosis and the incidence of breast and ovarian cancers, even among women who carry the same *BRCA1 *and *BRCA2 *mutation [6-8]. These risk estimates not only show that such women are at extremely high risk for developing breast cancer, but also illustrate that there is great variability in the time to breast cancer diagnosis among carriers. These observations are consistent with the hypothesis that the breast cancer risk in mutation carriers is modified by other genetic and/or environmental factors. There are published reports of genetic modifiers of cancer risk in mutation carriers (for example, variants in *AIB1 *in *BRCA1*; variants in *RAD51*, *FGFR2 *and *MAP3K1 *in *BRCA2*; and variants in *TNRC9 *in *BRCA1 *and *BRCA2*) [9-13].

One important pathway for cancer pathogenesis may be the insulin-like growth factor (IGF) signaling pathway, as it regulates both cellular proliferation and apoptosis. Extensive evidence from *in vivo *and *in vitro *model systems and human studies (reviewed in [14-16]) supports a major role for the IGF1 signaling pathway in breast cancer pathogenesis. Mammographic density, a strong risk factor for breast cancer, has been positively associated with the ratio of IGF1 to insulin-like growth factor binding protein (IGFBP)-3 in premenopausal women [17], and has been shown to modify the breast cancer risks in *BRCA1 *and *BRCA2 *mutation carriers [18]. BRCA1 has been shown to directly affect IGF1 signaling. In multiple experimental systems, including primary mammary tumors, cultured human cells, and Brca1-deficient mice, Shukla and colleagues showed that BRCA1 deficiency resulted in increased expression of insulin-like growth factor receptor substrate 1 (IRS1), insulin-like growth factor-1 receptor (IGF1R), IGFBP2, and increased levels of serum IGF1 [19]. In another study investigating IGF1R levels in breast tumors, there were significantly higher levels of IGF1R in tumors from *BRCA1 *mutation carriers as compared with noncarriers [20].

We hypothesized that genetic variation in IGF signaling will modify risk of breast cancer in women carrying deleterious mutations in *BRCA1 *and *BRCA2*. In the present study, we focused on investigating the association of variants in *IGF1, IGFBP1, IGFBP2*, *IGFBP5, IGF1R*, and *IRS1 *as potential disease modifiers in mutation carriers of *BRCA1 *and *BRCA2*.

## Materials and methods

### Participants

Women with germline, deleterious mutations in *BRCA1 *and *BRCA2 *were identified in 14 centers in the US, one center in Canada, and one center in Austria - including Baylor University Medical Center - Dallas, Beth Israel in Boston, City of Hope, Creighton University, Dana Farber, Fox Chase Cancer Center, Georgetown University, the Mayo Clinic, Medical University of Vienna, North Shore University Health System in Chicago, University of California, Los Angeles, University of California, Irvine, University of Chicago, University of Pennsylvania, and Women's College Hospital. The majority of subjects were recruited from the Medical University in Vienna, Creighton University in Nebraska, the University of Pennsylvania, and the University of California Irvine (previously at the University of Utah). All centers are part of the Modifiers and Genetics in Cancer consortium. All participants were enrolled under Institutional Review Boards or ethics committee approval at each participating site.

Women were participating in research studies or were either physician or self-referred to risk evaluation clinics for genetic testing, generally because of a strong family history of breast cancer and/or ovarian cancer. The current study is composed of a total of 1,665 adult, female mutation carriers, including 1,122 *BRCA1 *carriers (433 cases and 689 controls) and 543 *BRCA2 *carriers (238 cases and 305 controls). The *BRCA1 *and *BRCA2 *mutation status of all subjects was confirmed by direct mutation testing, with full informed consent under protocols approved by the human subjects review boards at each institution.

Women were eligible for entry into the study cohort if they tested positive for a known deleterious mutation in *BRCA1 *or *BRCA2*. Women with *BRCA1 *and *BRCA2 *variants of unknown functional significance were excluded. Women were excluded if they were missing information on year of birth, parity, menopausal status, and oral contraceptive use, or had been diagnosed with cancer more than 3 years prior to study entry. Information about invasive breast cancer, ovarian cancer, prophylactic mastectomy and prophylactic oophorectomy was obtained from medical records, and information on reproductive history and lifestyle habits was obtained by questionnaire.

### SNP genotyping

The 47 SNPs had been selected and genotyped in a previous case-control study of African-American women. Briefly, a minimal set of informative SNPs (tagging SNPs) had been chosen across each gene to mark the common genetic variation and to minimize the genotyping costs. Tagging SNP sets were selected using the TagSNPs program [21] from genotype data, downloaded directly from the National Institute of Environmental Health Sciences Environmental Genome Project [22]. For the data available at the time, it was not possible to select tagging SNPs for just a Caucasian population.

Genotyping was performed by the MGB Taqman probe Assay from Applied Biosystems Inc. (Foster City, CA USA) or the MGB Eclipse™ probe assay from Nanogen Inc. (San Diego, CA USA) for all SNPs. Primer and probe sequences are available from the authors on request.

Specifically, for the MGB Taqman probe assays, the reaction mix in a final volume of 5 μl included 10 ng genomic DNA, 4.5 pmol each primer, 1.25 pmol each probe, 1 × PCR reaction buffer (Qiagen, Gaithersburg, MD USA), 2 × Q solution (Qiagen), 500 pmol dNTP, and 0.15 units Qiagen DNA polymerase. PCR cycling included 55 cycles of a two-step PCR (95°C for 15 seconds, and 60°C for 1 minute) after an initial 2 minutes at 95°C. PCR plates were read on an ABI PRISM 7900 HT instrument for genotype assignment (Applied Biosystems Inc.).

Specifically, for the MGB Eclipse™ probe assays, the reaction mix in a final volume of 5 μl included 10 ng genomic DNA, 0.5 pmol limiting primer, 5 to 10 pmol excess primer, 1 pmol each probe, 1 × PCR reaction buffer (Qiagen), 2 × Q solution (Qiagen), 500 pmol dNTP, and 0.15 units Qiagen DNA polymerase. PCR cycling included 55 cycles of a three-step PCR (95°C for 10 seconds, 58°C for 20 seconds and 72°C for 20 seconds) after an initial 2 minutes at 95°C.

After completion of PCR, endpoint dissociation melting curves were generated on the ABI PRISM 7900 HT instrument by monitoring the fluorescence while heating the reactions from 30°C to 80°C at a 10% rate. An EclipseMeltMacro_v2.328 program (Nanogen Inc., San Diego, CA USA) was employed to assign the genotype from the dissociation curve data. Duplicates of 22 DNA samples and water controls were genotyped for quality control. The laboratory technician was blinded as to whether samples were duplicates, cases, or controls. The order of the DNA samples on 384-well plates was randomized in order to ensure balance in study conditions across covariates. Genotyping call rates ranged from 95% to 99% and duplicate concordance rates were higher than 99%.

### Determination of linkage disequilibrium blocks

We recalculated the linkage disequilibrium (LD) blocks for this study, primarily because we wanted the LD groups to reflect this predominantly non-Hispanic Caucasian cohort rather than the mixed sample from which the tagging SNPs were originally identified. SNPs were grouped according to their adjacent pairwise LD coefficient (*D'*). The coefficient was computed between all adjacent marker pairs within each candidate gene. In order to account for within-family correlation, multiple outputation [23] was used to estimate *D'*. In this case, a single member from each family was randomly sampled to create a single bootstrap sample, from which *D' *was computed. This process was repeated to obtain 200 bootstrap samples, yielding an empirical distribution of *D'*. An LD block was defined as a set of contiguous SNPs having *D' *values exceeding 0.90 between each contiguous pair of SNPs. The boundary of an LD block would be defined by a marker pair with *D' *≤ 0.9. The LD blocks for the SNPs within each gene are shown in Additional data file 1.

### Statistical analysis

Breast cancer rates were calculated as the observed number of breast cancers per total patient time at risk, and were standardized to the age distribution of the study cohort at the time of interview [24]. Subjects were considered at risk for breast cancer from birth until the first occurrence of breast cancer diagnosis, death, or loss to follow-up. In addition, subjects were censored in the event that they underwent a bilateral prophylactic surgery of the breasts more than 1 year preceding the diagnosis of breast cancer. Bilateral prophylactic surgery of the breasts occurring within 1 year of breast cancer was considered an event in order to avoid potential biases resulting from informative censoring.

Covariates that vary with time (ovarian cancer and prophylactic ovarian surgery) were treated as time dependent in the calculation of rates. A subject who was diagnosed with ovarian cancer therefore contributed time at risk in the non-ovarian cancer group prior to the diagnosis and then time at risk in the ovarian cancer group following the diagnosis. Because subjects were ascertained primarily from high-risk clinics, there was an oversampling of cases. In order to account for potential bias in cumulative risk estimates due to nonrandom sampling from the general population, Kaplan-Meier estimates of the cumulative probability of breast cancer diagnosis were computed using age-specific sampling weights for cases and controls. Sampling weights were obtained from Antoniou and colleagues [1].

Cox proportional hazards regression was used to model the time from birth to diagnosis of breast cancer. In this model, the hazard or instantaneous probability of breast cancer diagnosis is modeled as a function of the predictor covariates. The relative risk or hazard ratio (HR) is then interpreted for each covariate as the proportionate change in the instantaneous probability of diagnosis for two individuals, differing only by a single unit of that covariate. When analyzing LD blocks with multiple SNPs, an additive haplotype effect was assumed where the most common haplotype was used as the referent group for comparisons. When an LD block consisted of a single SNP, however, a general genetic model making no additivity assumption was used. In order to account for phase uncertainty in haplotype analysis, we used a two-step approximation to the semiparametric maximum likelihood estimator of Lin and Zeng [25]. Using this method, the expectation-maximization algorithm was used to compute posterior estimates of the probability of all potential haplotypes for a subject given their known genotype, and these probabilities were used to weight the individual's contribution to the partial likelihood. A similar approach has previously been applied to logistic regression models for analyzing case-control data and was shown to provide robust inference for relatively common haplotypes with little phase ambiguity [26]. In order to account for hierarchical clustering at the individual level (multiple records per individual were analyzed according to the number of potential diplotypes consistent with the individual's genotype) and at the family level (matched controls were often selected from the family of a case), the sandwich estimator of Lin and Wei [27] was used in combination with multiple outputation [23] to obtain robust variance estimates of haplotype associations.

All estimates were adjusted for birth cohort (to account for frequency matching of cases and controls), race/ethnicity, parity, and region of center (North American (US) vs. European). Ashkenazi Jewish individuals were considered a separate ethnicity because the carriers only had one of three founder mutations. Parity, prophylactic oophorectomy, and ovarian cancer status were treated as time-dependent covariates in the analysis, with these covariates updated at the time of childbirth. Beyond adjustment for birth cohort, no additional weighting for selection was employed. For LD blocks exhibiting significant associations with the time to breast cancer diagnosis, secondary analyses of individual SNPs making up the LD block were conducted. For all analyses, the proportional hazards assumption was examined by considering multiplicative interactions between each haplotype (or SNP) of interest and (log-transformed) time. No significant departures from the proportional hazards assumption were observed.

In total, the current analysis involves testing of 48 LD blocks, which is likely to result in an inflation of the family-wise type I error rate for the study if unadjusted critical values are used for assessing LD block significance. Noting that this analysis represents a first-stage in identifying variants in the IGF pathway that are associated with time to breast cancer diagnosis, we sought to control the family-wise type error rate at 15% in order to minimize the type II error rate, limiting the possibility of ruling out potentially important LD blocks from future investigation. Simulation was used to estimate the family-wise type I error rate, assuming a correlation of 0.75 across tests was assumed. Based upon 100,000 simulations it was estimated that an adjusted *P *value of 0.016 on any individual LD block test would result in a family-wise type I error rate of 15% for the study. An adjusted *P *< 0.016 was interpreted as a significant association.

## Results

**Figure 1:**
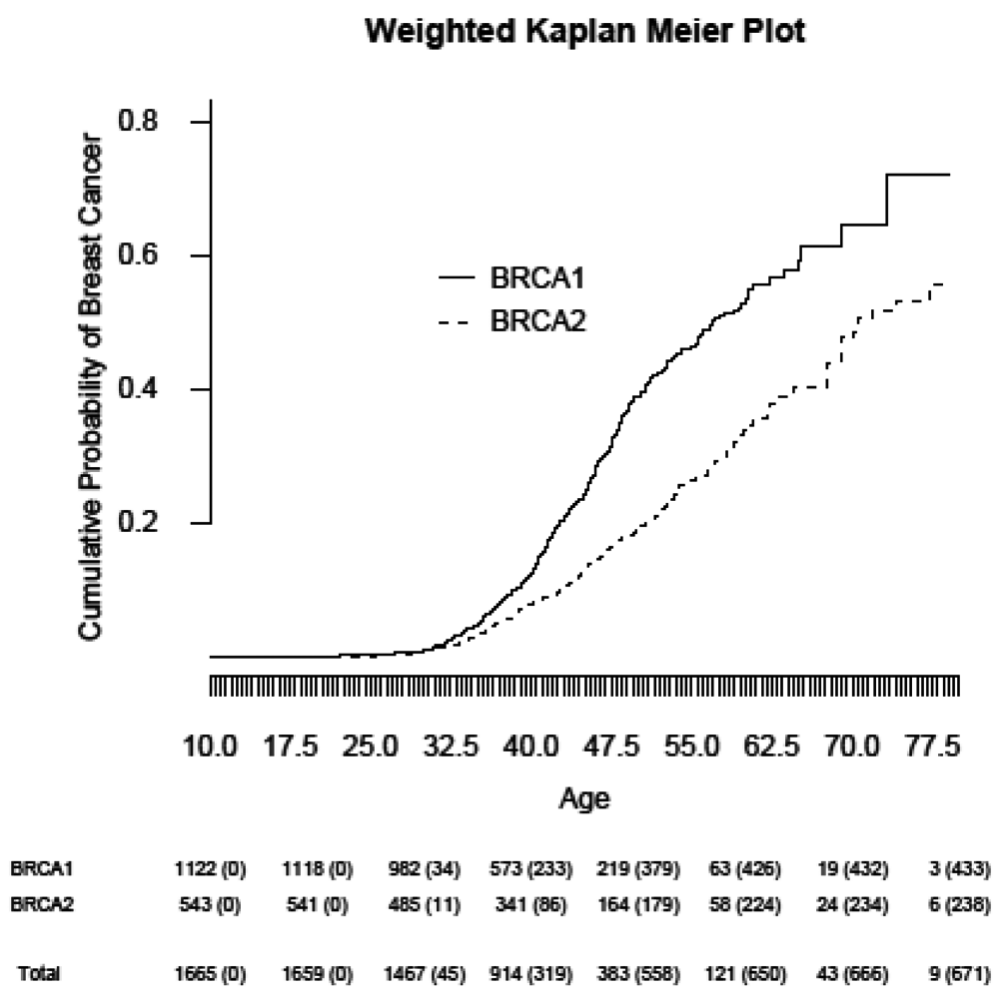
Kaplan-Meier estimates of the cumulative probability of breast cancer diagnosis by BRCA status.  Statistics in the lower portion of the plot represent the number of patients at risk (cumulative number of diagnoses) at each decade of life, ranging from 20 to 80 years. Estimates are weighted to account for oversampling of cases to controls [1].

The characteristics of the cases and the sites, and the observed incidence rate (per 1,000 women per year) of breast cancer diagnosis stratified by BRCA status are presented in Table 1. The presented rates have been externally standardized to the age distribution of the study cohort at the time of genetic testing. The study included 1,222 *BRCA1 *carriers (433 diagnosed with breast cancer) and 543 *BRCA2 *carriers (238 diagnosed with breast cancer). The age-standardized incidence rate of breast cancer diagnosis was estimated to be 26.94 per 1,000 per year in *BRCA1 *carriers (95% confidence interval (CI) = 19.79, 34.10) compared with 25.03 per 1,000 per year in *BRCA2 *carriers (95% CI = 18.71, 31.36). The majority of study subjects in both strata were White Caucasian (non-Jewish, non-Hispanic). Of the study subjects, 9.5% underwent bilateral prophylactic mastectomy (107/1,122 among *BRCA1 *carriers and 40/543 among *BRCA2 *carriers) and 39.4% underwent prophylactic bilateral salpingo-oophorectomy (449/1,122 among *BRCA1 *carriers and 207/543 among *BRCA2 *carriers). Figure 1 shows the estimated cumulative probabilities of breast cancer diagnosis in *BRCA1 *and *BRCA2 *carriers observed in the study. The median age at diagnosis was estimated to be 57.0 years (95% CI = 54.1, 62.2) among *BRCA1 *carriers and was 70.5 years (95% CI = 67.7, INF) among *BRCA2 *carriers.

**Table 1:** Participant characteristics and incidence of breast cancer diagnosis by BRCA status

	*BRCA1*			*BRCA2*		
	
Characteristic	*n*	Cases	**Incidence rate**^a^	*n*	Cases	**Incidence rate**^a^
Total	1,122	433	26.94 (19.79, 34.10)	543	238	25.03 (18.71, 31.36)
Race^b^						
Caucasian (non-Jewish, non-Hispanic)	774	283	26.72 (19.58, 33.86)	381	176	27.70 (20.63, 34.77)
African American	29	14	39.37 (28.13, 50.61)	13	6	25.28 (18.47, 32.09)
Jewish	245	98	24.08 (17.95, 30.22)	119	43	17.39 (13.19, 21.60)
Caucasian Hispanic	35	17	43.97 (30.46, 57.47)	9	5	33.07 (22.50, 43.65)
Other	31	16	38.66 (28.28, 49.05)	19	6	20.68 (15.61, 25.74)
Ovarian cancer						
Yes	128	26	20.86 (11.70, 30.01)	30	5	18.90 (8.84, 28.96)
No	994	407	27.78 (20.46, 35.10)	513	233	25.43 (19.10, 31.77)
Prophylactic ovarian surgery						
Yes before breast cancer	282	44	24.88 (17.53, 32.23)	108	18	27.64 (19.07, 36.21)
Yes after breast cancer	167	167	--	99	99	--
No bilateral prophylactic oophorectomy	671	221	28.07 (20.72, 35.42)	336	121	25.29 (18.93, 31.64)
Clinic Site						
Medical University Vienna	204	84	39.26 (29.31, 49.21)	62	37	28.19 (20.83, 35.55)
Beth Israel	8	4	30.58 (22.39, 38.76)	15	6	125.51 (35.71, 215.31)
Baylor University Medical Center --Dallas	14	10	69.46 (50.29, 88.63)	1	1	14.59 (10.21, 18.97)
City of Hope	56	25	43.30 (31.79, 54.81)	28	17	54.17 (40.17, 68.18)
Creighton	155	65	28.49 (21.39, 35.59)	40	23	55.87 (42.83, 68.91)
Dana Farber	88	41	36.22 (27.27, 45.18)	32	11	27.20 (21.04, 33.37)
NorthShore University Health System	35	16	26.55 (19.62, 33.47)	21	9	17.77 (13.57, 21.97)
Fox Chase Cancer Center	40	10	14.41 (9.97, 18.86)	28	9	18.18 (13.32, 23.04)
Georgetown University	42	13	21.35 (16.23, 26.47)	16	3	21.64 (16.24, 27.05)
University of California, Los Angeles	43	18	36.95 (25.09, 48.81)	17	7	13.62 (10.03, 17.21)
Mayo Clinic	60	17	18.57 (14.53, 22.61)	31	10	30.38 (24.01, 36.75)
University of Texas Health Science Center at San Antonio	35	17	40.18 (27.33, 53.02)	32	13	11.67 (9.00, 14.34)
University of Chicago	34	15	52.84 (36.66, 69.02)	18	9	18.73 (14.47, 22.98)
University of Pennsylvania	147	56	24.08 (17.37, 30.78)	92	44	51.62 (43.58, 59.66)
University of Utah^c^	115	30	14.85 (10.70, 19.00)	87	27	27.54 (19.36, 35.72)
Women's College Hospital, Toronto	46	12	16.24 (11.72, 20.76)	23	12	44.56 (33.11, 56.02)
Age^d^			44.7 ± 11.2			48.1 ± 13

^a^Data presented as incidence per 1,000 women per year (95% confidence interval). Rates have been externally standardized to the age distribution of the study cohort at the time of genetic testing. ^b^Five subjects missing race information. ^c^Now at University of California, Irvine. ^d^Data presented as mean ± standard deviation.

### IGF binding proteins IGFBP1, IGFBP2, and IGFBP5

**Figure 2:**
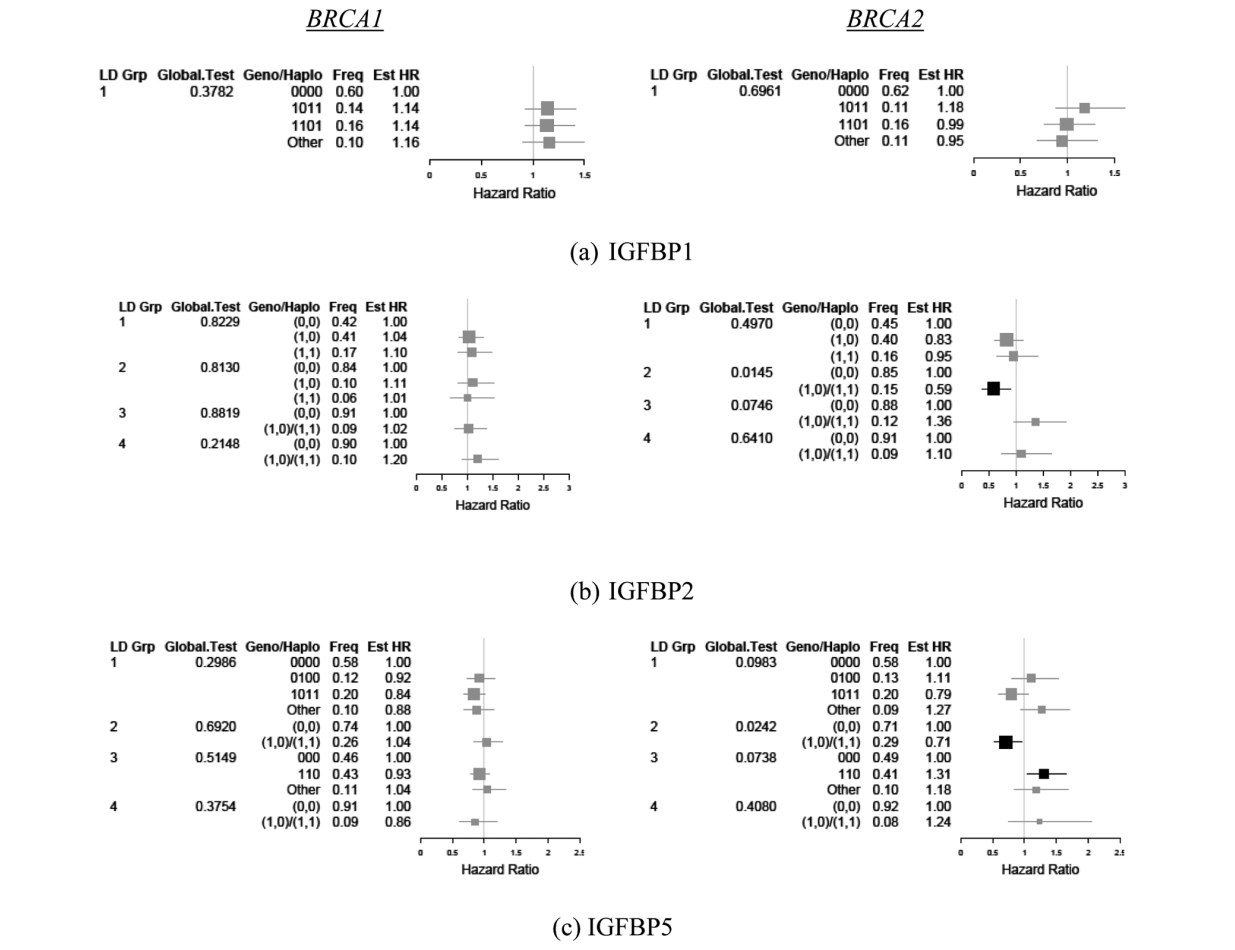
Haplotype presence for insulin-like growth factor binding proteins.  Estimated hazard ratios (Est HR) associated with haplotype presence for **(a) **insulin-like growth factor binding protein (IGFBP)-1, **(b) **IGFBP2, and **(c) **IGFBP5. Linkage blocks were defined as pairwise linkage disequilibrium coefficient *D*' ≥ 0.90. Estimates were stratified by BRCA status (left column, *BRCA1*; right column, *BRCA2*) and adjusted for birth cohort and ethnicity as well as first pregnancy, prophylactic oophorectomy, and diagnosis of ovarian cancer as time-dependent covariates. LD Grp, linkage disequilibrium group; Geno/Haplo, genotype/haplotype; Freq, frequency.

Figure 2 presents the estimated HR for time to diagnosis by LD block within each of the IGFBPs, and the BRCA status after adjustment for covariates (described in Materials and methods). For *BRCA1 *carriers, no significant associations were observed for the three IGF binding genes. Among *BRCA2 *carriers, one LD block in *IGFBP2 *showed significance in the hazard for diagnosis. For *IGFBP2 *LD block 2 (defined by a single SNP rs9341134), women with at least one variant allele were estimated to experience a 41% lower risk of diagnosis when compared with women with no variant alleles (HR = 0.59; 95% CI = 0.39, 0.90; unadjusted global *P *= 0.0145). For *IGFBP5 *LD block 2 (defined by a single SNP rs2241193), women with at least one variant allele were estimated to experience a 29% lower risk of diagnosis when compared with women with no variant alleles (HR = 0.71; 95% CI = 0.53, 0.96; unadjusted global *P *= 0.0242).

### Insulin-like growth factor receptor substrate 1 and insulin-like growth factor 1

**Figure 3:**
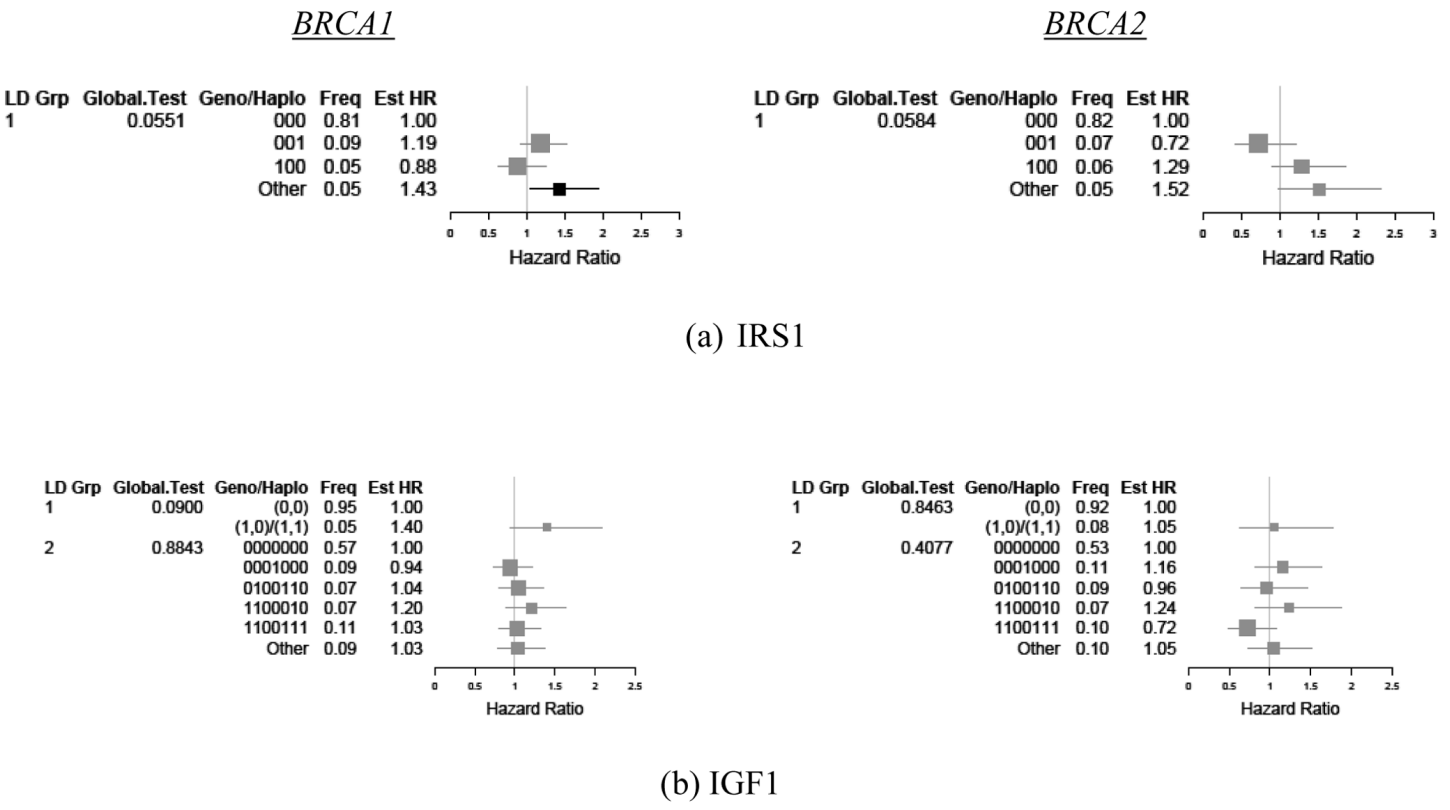
Haplotype presence for insulin-like growth factor receptor substrate 1 and insulin-like growth factor 1.  Estimated hazard ratios (Est HR) associated with haplotype presence for **(a) **insulin-like growth factor receptor substrate 1 (IRS1) and **(b) **insulin-like growth factor 1 (IGF1). Linkage blocks were defined as in Figure 2 (pairwise linkage disequilibrium coefficient *D*' ≥ 0.90). Estimates were stratified by BRCA status (left column, *BRCA1*; right column, *BRCA2*) and adjusted for birth cohort and ethnicity as well as first pregnancy, prophylactic oophorectomy, and diagnosis of ovarian cancer as time-dependent covariates. LD Grp, linkage disequilibrium group; Geno/Haplo, genotype/haplotype; Freq, frequency.

Estimated HRs for the haplotypes of *IRS1 *are shown in Figure 3a. Among *BRCA1 *carriers, the global LD block test for *IRS1 *was not significant (unadjusted global *P *= 0.0551). Relative to the referent haplotype, however, individuals with haplotypes homozygous for the common variant (excluding haplotypes 001 and 100) were estimated to have a 43% (CI = 1.06, 1.95; *P *= 0.02) higher risk of breast cancer diagnosis.

We then investigated the HRs for the three SNPs within the LD block to determine whether the observed haplotype associations were attributable to particular SNPs (Table 2). For SNPs rs13306465 and rs1801123, individuals carrying at least one variant allele experienced a 44% (HR = 1.44; 95% CI = 1.07, 1.94; unadjusted *P *= 0.0165) and 37% (HR = 1.37; 95% CI = 1.11, 1.69; unadjusted *P *= 0.0033) higher risk of breast cancer relative to wild-type carriers, respectively. There was no individual association of the rs1801278 (G972R) SNP and risk. For the single *IRS1 *LD block, a similar, but nonsignificant HR of 1.52 (95% CI = 0.99, 2.32; unadjusted *P *= 0.055) was observed in *BRCA2 *carriers.

**Table 2:** Single SNP analysis results within significant linkage disequilibrium blocks for *BRCA1 *carriers

	LD block^a^	SNP	Genotype	*n*	Hazard ratio	95% confidence interval	*P *for trend
IRS1	1	rs1801278	GG	990			
			GA, AA	115	0.82	0.58, 1.17	
	1	rs13306465	GG	999			
			GA, AA	97	1.44	1.07, 1.94	
	1	rs1801123	AA	823			
			AG, GG	279	1.37	1.11, 1.69	
IGF1R	11	rs8038415	CC	281			
			CT	536	1.11	0.88, 1.41	
			TT	270	1.40	1.07, 1.83	0.015
	11	rs17847201	GG	350			
			GA	581	0.87	0.69, 1.10	
			AA	154	0.77	0.56, 1.05	0.091

IRS1, insulin-like growth factor receptor substrate 1; IGF1R, insulin-like growth factor-1 receptor. ^a^Linkage disequilibrium (LD) block was defined by pairwise LD coefficient *D*' ≥ 0.90.

For *IGF1*, no significant associations were found for either *BRCA1 *or *BRCA2 *carriers (Figure 3b).

### Insulin-like growth factor-1 receptor

**Figure 4:**
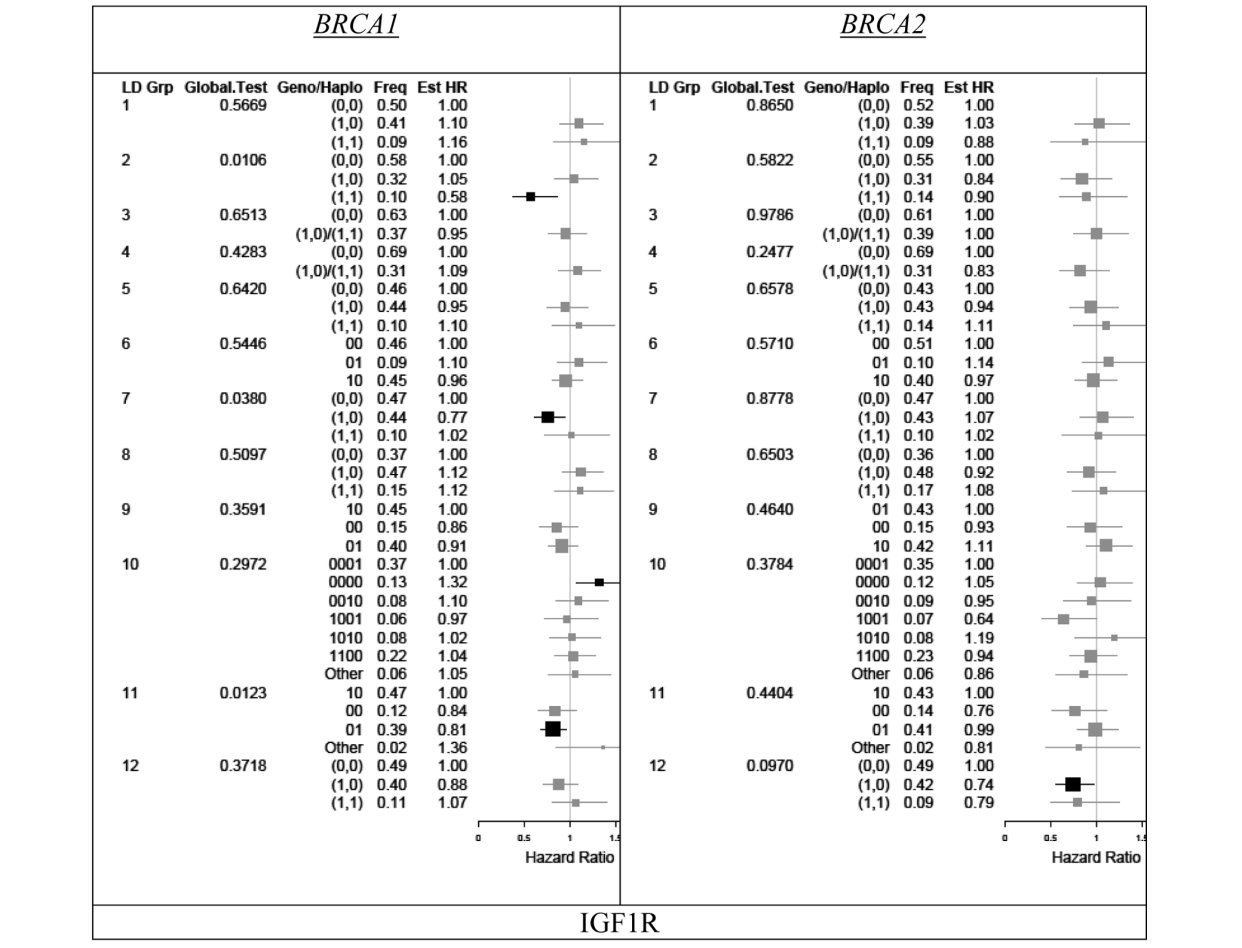
Haplotype presence for insulin-like growth factor-1 receptor.  Estimated hazard ratios (Est HR) associated with haplotype presence for insulin-like growth factor-1 receptor (IGF1R). Linkage blocks were defined as in Figure 2 (pairwise linkage disequilibrium coefficient *D*' ≥ 0.90). Estimates were stratified by BRCA status (left column, *BRCA1*; right column, *BRCA2*) and adjusted for birth cohort and ethnicity as well as first pregnancy, prophylactic oophorectomy, and diagnosis of ovarian cancer as time-dependent covariates. LD Grp, linkage disequilibrium group; Geno/Haplo, genotype/haplotype; Freq, frequency.

Figure 4 shows HR estimates for the 12 LD blocks genotyped in *IGF1R*. For *BRCA1 *carriers, significant associations were found between LD block 2 (SNP rs2715415) and LD block 11 and the risk of breast cancer diagnosis (unadjusted global *P *values corresponding to a test of homogeneity of risk within the LD blocks were 0.011 for LD block 2 and 0.012 for LD block 11). While qualitatively consistent associations were also observed among *BRCA2 *carriers, they were not significant. After investigation in *BRCA1 *carriers of the individual SNPs within LD block 11 (Table 2), the only SNP that was significantly associated with risk was rs8038415 - in which individuals homozygous for the variant allele were estimated to experience a 40% higher risk of breast cancer diagnosis (unadjusted *P *= 0.014, with *P *for trend = 0.015).

## Discussion

The IGF pathway plays essential roles in regulating cell proliferation, differentiation, and apoptosis. It is a key factor in the development and progression of breast cancer, based on evidence from more than 1,100 published papers, ranging from *in vivo *and *in vitro *studies in humans and mice to epidemiologic studies (reviewed in [14-16]). This is the first study to investigate the role of genetic variants in IGF signaling as modifiers of breast cancer risk in women who carry deleterious mutations in *BRCA1 *and *BRCA2*. We investigated only a small number of the genes involved in IGF signaling. We found significant HRs associated with genetic variants in *IGF1R *and *IRS1 *in *BRCA1 *carriers, and in *IGFBP2 *in *BRCA2 *carriers. No other significant associations in the studied genes were identified.

There have been a limited number of epidemiologic studies of the association of sporadic breast cancer risk and genetic variation in genes in the IGF pathway. For IGF1, the primary ligand for the IGF1R, there have been inconsistent reports of associations with breast cancer risk with reports showing significant associations [28,29] and no associations [30-35]. The inconsistent results may be due to differences in genetic variants examined in the genes and/or in study design (for example, restriction to postmenopausal or premenopausal breast cancers). Several studies of SNPs in *IGFBP1 *reported no association with breast cancer, similar to what we observed for *BRCA1 *and *BRCA2 *mutation carriers [28,35,36].

The IGFBPs serve as growth modulators, both independently and as regulators of IGFs [37-39]. IGFBP5 and IGFBP2 are overexpressed in breast cancer tissues [40,41], and are involved in apoptosis [42-44]. In a study of African Americans, with replication in Nigerians, we reported significant associations of SNPs within the *IGFBP2 *to *IGFPB5 *region and the risk of breast cancer [45]. These two genes are in a tail-to-tail configuration separated by only 10 kb on chromosome 2q, so it is possible the same underlying causal variation results in an association with both genes. In the present study, we report a significant association of *IGFBP2 *SNP rs9341134, also observed in the previous study [45], and marginally significant associations with variants in *IGFBP5*. Resequencing is needed to try to identify the actual causal variant. Another piece of evidence that this region may be associated with breast cancer is the association of SNP rs13387042 with a 1.2-fold increased risk in breast cancer, reported in a deCODE genome-wide association study [46] - with replication by the Cancer Genetic Markers of Susceptibility project (odds ratio = 1.2) [47], by the Breast Cancer Association Consortium (odds ratio = 1.14) [48], and by the Consortium of Investigators of Modifiers of *BRCA1 *and *BRCA2 *(HR = 1.14 and HR = 1.18 for *BRCA1 *and *BRCA2 *carriers, respectively) [49]. It is hypothesized that this SNP may act as a long-range regulatory element on expression of IGFBP2 or IGFBP5 [46].

Of the genes examined, only genetic variants in IGF1R and its adaptor protein IRS1 were associated with risk of breast cancer in *BRCA1 *carriers. IGF1R has both mitogenic and antiapoptotic roles in tumor development via signaling through the phosphatidylinositol-3-kinase and mitogen-activated protein kinase pathways [50], with its adaptor protein IRS1 critical in activating the downstream pathways. Both IGF1R overexpression and IRS1 overexpression have been associated with breast cancer development, and IGF1R is overexpressed in a majority of breast tumors [51]. Interestingly, BRCA1 directly affects IGF1 signaling. In multiple experimental systems including primary mammary tumors, cultured human cells, and Brca1-deficient mice, Shukla and colleagues showed that BRCA1 deficiency resulted in increased expression of IRS1, IGF1R and IGFBP2, and increased levels of serum IGF1 [19]. In another study investigating IGF1R levels in breast tumors, there were significantly higher levels of IGF1R in tumors from *BRCA1 *mutation carriers as compared with noncarriers [20].

In a series of experiments co-transfecting cell lines with IGF1R promoter constructs driving luciferase reporter genes, and a BRCA1 expression vector, it was shown that BRCA1 suppressed IGF1R promoter activity in a dose-dependent manner [52], through preventing binding of Sp1 to the IGF1R promoter, thus reducing transcription [52,53]. As demonstrated using western blots, wild-type BRCA1 was able to induce a large reduction in endogenous IGF1R levels [20]. In addition to its interaction with the IGF1R, BRCA1 interacts directly with the IRS1 promoter to inhibit its activity [19]. With induction of BRCA1, the authors observed a twofold and threefold decrease of IRS1 mRNA and protein levels, respectively, as well as a decrease in the phosphorylation level of AKT, a downstream target of IGF1R and IRS1 [19].

Based on these experiments, there is strong evidence that mutant forms of *BRCA1 *cause increased IGF1R activation, leading to a decrease in apoptosis and a concomitant increased survival of malignant cells, which then can proliferate. There is therefore a strong rationale for why genetic variation in IGF1R and IRS1 would be important in breast cancer risk in *BRCA1 *carriers. Experimental studies have not been published for BRCA2 to demonstrate whether there is a similar effect on transcriptional regulation.

As noted above, this is the first study to investigate the role of genetic variants in IGF signaling as modifiers of breast cancer risk in women who carry deleterious mutations in *BRCA1 *and *BRCA2*. While the study does provide an important first step in identifying potential genetic modifiers of risk among *BRCA1 *and *BRCA2 *carriers, it does suffer some limitations. First, although the IGF pathway was hypothesized *a priori *as a source for potential modifiers, multiple LD blocks were considered for association testing and such testing could lead to inflation of the overall type I error rate for the study. With this said, we only studied a small number of the genes in IGF signaling that we deemed *a priori *would potentially play a role in the time to diagnosis. Further, the goal of the current research was to generate hypotheses based upon the results from this well-defined set of genes, and it is our intention to further validate these results using an independent sample. As with all observational studies, there is the potential for selection bias and unmeasured confounding. We have, however, adjusted for those environmental factors that previous research has shown to most highly influence the risk of breast cancer diagnosis within this cohort, thus lowering the potential for unadjusted confounding.

We and others have investigated putative risk factors, and a number of published studies have implicated candidate genes (for example, *AIB1 *in *BRCA1*, *RAD51 *in *BRCA2*) and SNPs in *FGFR2*, *MAP3K1*, *TNRC9*, *LSP1*, and 2q35 previously identified from genome-wide association studies of breast cancer as modifiers of breast cancer or ovarian cancer penetrance in women who carry germline *BRCA1 *or *BRCA2 *mutations [9-13,49]. Our results suggest that variation in genes in IGF signaling also modify breast cancer penetrance in *BRCA1 *and *BRCA2 *carriers.

## Conclusions

The present study is the first to investigate the role of genetic variation in IGF signaling and breast cancer risk in women carrying deleterious mutations in *BRCA1 *and *BRCA2*. We identified significant associations for variants in *IGF1R *and *IRS1 *for *BRCA1 *carriers and for variants in *IGFBP2 *for *BRCA2 *carriers. Given the known interaction of BRCA1 and IGF signaling, and specifically the regulation of IRS1 and IGF1R by BRCA1, further replication and identification of causal mechanisms are needed to validate and better understand these associations.

## Abbreviations

CI: confidence interval; *D'*: pairwise linkage disequilibrium coefficient; HR: hazard ratio; IGF: insulin-like growth factor; IGF1R: insulin-like growth factor-1 receptor; IGFBP: insulin-like growth factor binding protein; IRS1: insulin-like growth factor receptor substrate 1; LD: linkage disequilibrium; PCR: polymerase chain reaction; SNP: single nucleotide polymorphism.

## Competing interests

The authors declare that they have no competing interests.

## Authors' contributions

SLN designed and conceived of this work, was responsible for the genotyping and collection of all data, interpreted the results, and drafted and wrote the manuscript. SB was responsible for analysis of the data, interpreted the results, prepared tables and figures, and drafted and edited the manuscript. YCD was responsible for performing and overseeing the genotyping and quality control of the genotyping, and edited the manuscript. CFS, GP, HTL, KLN, TRR, JEG, FC, JW, SAN, PAG, MBD, AG, CI, OIO, GT, WSR, NT, and JLB all provided the *BRCA1 *and *BRCA2 *mutation carriers, including samples and data, and reviewed and edited the manuscript. DLG was responsible for developing the statistical analysis and overseeing the programming and analysis of SB, interpreted the results, and drafted and wrote the manuscript. All authors read and approved the final manuscript.

## Supplementary Material

Additional file 1 Word file containing a table that lists the LD blocks for the SNPs within each gene and the minor allele frequencies (MAF) for each SNP Click here for file
