# Supplementary material for: Genetic variation in insulin-like growth factor signaling genes and breast cancer risk among BRCA1 and BRCA2 carriers
Source: Breast Cancer Res. 2009 Oct 20;11(5):R76. doi: 10.1186/bcr2414 (PMC2790858; doi:10.1186/bcr2414)
Supplement: Additional file 1 — Word file containing a table that lists the LD blocks for the SNPs within each gene and the minor allele frequencies (MAF) for each SNP [file bcr2414-S1.docx]

**Supplementary Table. SNPs and their Minor Allele Frequencies (MAF)**

|  |  |  | BRCA1 | |  | BRCA2 | |
| --- | --- | --- | --- | --- | --- | --- | --- |
| Gene | LD Block | SNP | Unaffected | Cases |  | Unaffected | Cases |
| IGFBP1 | 1 | rs3793344 | 0.37 | 0.422 |  | 0.352 | 0.363 |
|  | 1 | rs2854843 | 0.161 | 0.178 |  | 0.164 | 0.182 |
|  | 1 | rs9658223 | 0.145 | 0.167 |  | 0.118 | 0.129 |
|  | 1 | rs4619 | 0.337 | 0.385 |  | 0.314 | 0.349 |
|  |  |  |  |  |  |  |  |
| IGFBP2 | 1 | rs7603372 | 0.372 | 0.383 |  | 0.363 | 0.344 |
|  | 2 | rs9341134 | 0.105 | 0.113 |  | 0.118 | 0.065 |
|  | 3 | rs9341145 | 0.047 | 0.058 |  | 0.057 | 0.072 |
|  | 4 | rs9341227 | 0.05 | 0.054 |  | 0.036 | 0.056 |
|  |  |  |  |  |  |  |  |
| IGFBP5 | 1 | rs3770472 | 0.254 | 0.235 |  | 0.25 | 0.228 |
|  | 1 | rs11575130 | 0.118 | 0.125 |  | 0.134 | 0.13 |
|  | 1 | rs11575134 | 0.233 | 0.206 |  | 0.228 | 0.209 |
|  | 1 | rs10932673 | 0.267 | 0.241 |  | 0.249 | 0.252 |
|  | 2 | rs2241193 | 0.131 | 0.138 |  | 0.174 | 0.136 |
|  | 3 | rs741384 | 0.498 | 0.499 |  | 0.45 | 0.489 |
|  | 3 | rs11575161 | 0.434 | 0.437 |  | 0.389 | 0.443 |
|  | 3 | rs7565131 | 0.065 | 0.065 |  | 0.067 | 0.067 |
|  | 4 | rs6746360 | 0.047 | 0.04 |  | 0.042 | 0.047 |
|  | 4 | rs11575213 | 0.047 | 0.042 |  | 0.048 | 0.043 |
|  |  |  |  |  |  |  |  |
| IRS1 | 1 | rs13306465 | 0.041 | 0.057 |  | 0.033 | 0.06 |
|  | 1 | rs1801123 | 0.123 | 0.159 |  | 0.109 | 0.13 |
|  | 1 | rs1801278 | 0.058 | 0.047 |  | 0.062 | 0.056 |
|  |  |  |  |  |  |  |  |
| IGF1 | 1 | rs5742612 | 0.024 | 0.033 |  | 0.036 | 0.049 |
|  | 2 | rs2195240 | 0.247 | 0.251 |  | 0.227 | 0.221 |
|  | 2 | rs17879400 | 0.269 | 0.293 |  | 0.306 | 0.292 |
|  | 2 | rs17882122 | 0.011 | 0.022 |  | 0.019 | 0.026 |
|  | 2 | rs1549593 | 0.122 | 0.129 |  | 0.14 | 0.126 |
|  | 2 | rs1520220 | 0.202 | 0.223 |  | 0.232 | 0.221 |
|  | 2 | rs6220 | 0.285 | 0.316 |  | 0.326 | 0.327 |
|  | 2 | rs6219 | 0.12 | 0.116 |  | 0.112 | 0.098 |
|  |  |  |  |  |  |  |  |
| IGF1R | 1 | rs2684810 | 0.286 | 0.302 |  | 0.286 | 0.282 |
|  | 2 | rs2715415 | 0.272 | 0.239 |  | 0.303 | 0.288 |
|  | 3 | rs7168671 | 0.216 | 0.2 |  | 0.221 | 0.21 |
|  | 4 | rs2139924 | 0.165 | 0.184 |  | 0.168 | 0.175 |
|  | 5 | rs2684808 | 0.313 | 0.329 |  | 0.357 | 0.354 |
|  | 6 | rs2684806 | 0.448 | 0.441 |  | 0.398 | 0.397 |
|  | 6 | rs2684805 | 0.088 | 0.091 |  | 0.083 | 0.112 |
|  | 7 | rs4966044 | 0.379 | 0.404 |  | 0.405 | 0.404 |
|  | 8 | rs17847202 | 0.326 | 0.298 |  | 0.309 | 0.318 |
|  | 9 | rs2229765 | 0.438 | 0.466 |  | 0.434 | 0.402 |
|  | 9 | rs2684799 | 0.408 | 0.391 |  | 0.429 | 0.429 |
|  | 10 | rs2684793 | 0.407 | 0.394 |  | 0.415 | 0.442 |
|  | 10 | rs1568502 | 0.238 | 0.231 |  | 0.228 | 0.263 |
|  | 10 | rs2684790 | 0.161 | 0.169 |  | 0.183 | 0.159 |
|  | 10 | rs17847194 | 0.456 | 0.422 |  | 0.429 | 0.439 |
|  | 11 | rs8038415 | 0.483 | 0.517 |  | 0.478 | 0.421 |
|  | 11 | rs17847201 | 0.417 | 0.396 |  | 0.401 | 0.457 |
|  | 12 | rs3833015 | 0.311 | 0.313 |  | 0.32 | 0.271 |
